# E2F1 induces *TINCR* transcriptional activity and accelerates gastric cancer progression via activation of *TINCR/*STAU1*/*CDKN2B signaling axis

**DOI:** 10.1038/cddis.2017.205

**Published:** 2017-06-01

**Authors:** Tong-Peng Xu, Yan-Fen Wang, Wei-Liang Xiong, Pei Ma, Wen-Yu Wang, Wen-Ming Chen, Ming-De Huang, Rui Xia, Rong Wang, Er-Bao Zhang, Yan-Wen Liu, Wei De, Yong-Qian Shu

**Affiliations:** 1 Department of Oncology, The First Affiliated Hospital of Nanjing Medical University, Nanjing, P.R. China; 2 Department of Pathology, The Affiliated Hospital of Yangzhou University, Yangzhou University, Yangzhou, P.R. China; 3 State Key Laboratory of Microbial Metabolism, and School of Life Science and Biotechnology, Shanghai Jiaotong University, Shanghai, P.R. China; 4 Department of Oncology, Jining No.1 People’s Hospital, Jining City, China; 5 Department of Medical Oncology, Huai'an First People's Hospital, Nanjing Medical University, Huai'an City, P.R. China; 6 Department of Medical Laboratory, Nanjing Chest Hospital, Nanjing, P.R. China; 7 Department of Biochemistry and Molecular Biology, Nanjing Medical University, Nanjing, P.R. China

## Abstract

Recent evidence indicates that E2F1 transcription factor have pivotal roles in the regulation of cellular processes, and is found to be dysregulated in a variety of cancers. Long non-coding RNAs (lncRNAs) are also reported to exert important effect on tumorigenesis. E2F1 is aberrantly expressed in gastric cancer (GC), and biology functions of E2F1 in GC are controversial. The biological characteristics of E2F1 and correlation between E2F1 and lncRNAs in GC remain to be found. In this study, integrated analysis revealed that E2F1 expression was significantly increased in GC cases and its expression was positively correlated with the poor pathologic stage, large tumor size and poor prognosis. Forced E2F1 expression promotes proliferation, whereas loss of E2F1 function decreased cell proliferation by blocking of cell cycle in GC cells. Mechanistic analyses indicated that E2F1 accelerates GC growth partly through induces *TINCR* transcription. *TINCR* could bind to STAU1 (staufen1) protein, and influence *CDKN2B* mRNA stability and expression, thereby affecting the proliferation of GC cells. Together, our findings suggest that E2F1*/TINCR/*STAU1/CDKN2B signaling axis contributes to the oncogenic potential of GC and may constitute a potential therapeutic target in this disease.

Gastric cancer (GC) is still one of the most significant health problems in the world with particularly high frequencies in East Asia.^1^ The roles of genetic dysregulation, epigenetic changes and signaling pathways involved in cancer have recently been studied intensively.^2, 3, 4^ The use of gene expression data to predict carcinogenesis holds promise in GC diagnosis and prognosis. Thus, novel prognostic and diagnostic factors that are associated with GC progression would be of great clinical relevance.

The E2F transcription factors are key participants in a number of cellular events such as cell cycle, DNA synthesis or nuclear transcription. The E2F family of transcription factors is composed of activator (E2f1-3a) and repressor (E2f3b, 4–8) factors and is predominantly regulated by the Rb family of proteins (Rb, p107 and p130),^5, 6^ and the activating E2F transcription factors E2F1, E2F2 and E2F3 are central to regulation of the cell cycle genes.^7^ E2F1 is the most thoroughly investigated member of the E2F family in human malignancies. E2F1 has pivotal roles in tumor progression by modulation of both coding and non-coding transcripts,^8, 9^ and was reported to act as oncogenes or tumor suppressors to modulate tumorigenesis depending on different cell context.^8, 10, 11^ Accumulating evidence revealed E2F1 exert important effect on GC progression; however, the biology functions remain argued.^12, 13, 14^

*TINCR*, a long non-coding RNA (lncRNA) producing a 3.7-kb transcript, was first reported to bind to staufen1 (STAU1) protein and mediate differentiated mRNA stabilization.^15^ STAU1 protein is a double-stranded RNA-binding protein, and has various roles in gene expression. STAU1 binds to an STAU1-binding site in the 3′-untranslated region (3′-UTR) of its target mRNAs inducing mRNA degradation, which is termed STAU1-mediated mRNA decay (SMD).^16^ SMD is a translation-dependent mechanism that occurs when STAU1, together with the nonsense-mediated mRNA decay factor UPF1, is bound sufficiently downstream of a termination codon.^16^ Recently, we found that the expression of *TINCR* was elevated at the mRNA levels in GC cells and tissues and the upregulation of *TINCR* is induced by the transcription factor SP1.^17^ *TINCR* regulates cell growth, cell cycle progression by affecting *KLF2* mRNA stability via SMD.^17^

Here we report a novel pathway involved in E2F1 and *TINCR* in tumor development and GC cell growth. In this study, we found that: (a) E2F1 could promote GC proliferation and cell cycle progression; (b) patients with high E2F1 expression in their GC cells have a poor prognosis; (c) E2F1 could induce *TINCR* transcription activation; and (d) *TINCR* forces cell growth, cell cycle progression by affecting *CDKN2B* mRNA stability via SMD.

## Results

### E2F1 is overexpressed in GC tissues and cell lines, and upregulation of E2F1 indicate poor outcome of GC

**Figure 1:**
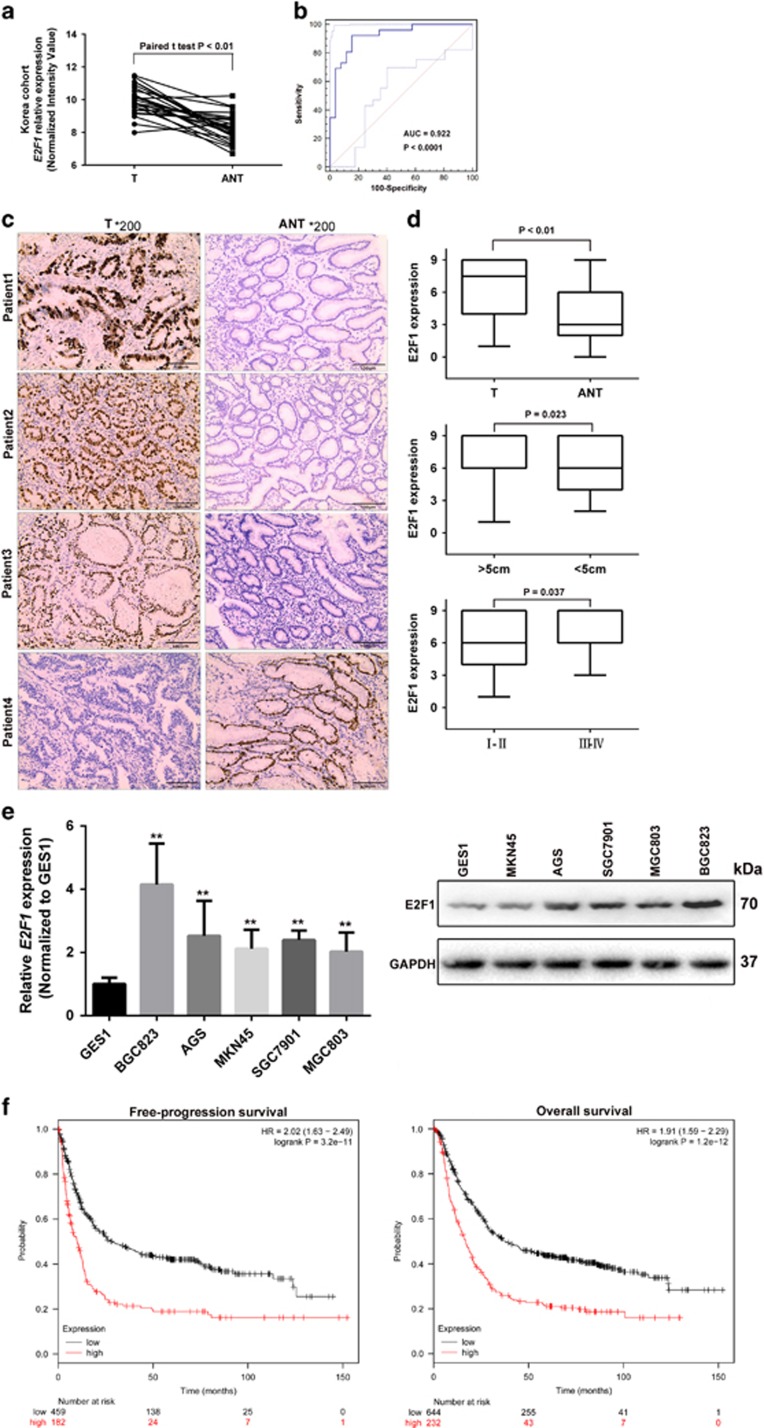
E2F1 is overexpressed in GC tissues and cell lines. (**a**) Analysis of E2F1 mRNA expression in GC and paired ANTs based on GSE51575 microarray database. (**b**) The ROC curve for prediction of GC based on E2F1 expression level in GSE51575, using corresponding adjacent non-tumorous tissues as a control. (**c**) IHC analysis of E2F1 protein expression in GC tumor tissues (T) and paired ANTs. Pictures of representative areas were presented at different staining intensities in ANT and T. (**d**) Analysis of the expression pattern of E2F1 in gastric tissues detected by IHC. Stages I–IV, TNM stages. Statistical analyses were performed using Student’s paired *t*-test and one-way ANOVA. (**e**) Real-time RT-PCR and western blot analyzed the expression of E2F1 in a series of human GC cell lines and normal gastric epithelial cell line (GES-1). (**f**) Kaplan–Meier survival plots demonstrating the good prognostic effect of E2F1 upregulation correlated with a worse FPS and OS in GC patients (*n*=876)

To investigate the role of E2F1 in the progression of human GC, a human microarray data sets (GSE51575) (26 paired cancer and noncancer tissues) was obtained to analyze E2F1 mRNA expressed between GC and paired non-tumor tissues. The result showed that E2F1 mRNA was 3.34-fold higher in gastric tumor tissues (T) compared with paired adjacent normal tissues (ANTs) (Figure 1a). We plotted a receiver operating characteristic (ROC) curve with the non-tumorous tissues adjacent to the tumor tissues as a control based on GSE51575 database. The cutoff value for predicting GC tissues from normal tissues was 8.91 (normalized intensity value). The area under the ROC curve (AUC) was 0.922 (95% confidence interval (CI)=0.813–0.978, *P*<0.0001), with the sensitivity and specificity were 0.923 and 0.846, respectively (Figure 1b). We further confirmed E2F1 expression levels between clinical gastric tumors (T) and paired ANTs from 80 cases of GC patients by immunohistochemistry (IHC) in our cohort. Our results showed that E2F1 was predominantly located in the nucleus of GC cells (Figure 1c). E2F1 expression found in GC tissues was significantly higher than in their adjacent tissues (*P*<0.001, Figure 1d and Supplementary Table S2). We also confirmed that E2F1 expression was significantly increased in larger tumors (*P*=0.023) and advanced TNM stages (*P*=0.037, Figure 1d). We further evaluated the expression levels of E2F1 in GC cell lines. The results showed that the expression levels of E2F1 were significantly increased in all tumourigenic GC cell lines than that in non-tumourigenic cell lines (Figure 1e). In addition, E2F1 expression is positively associated with FP (free progression) (hazard ratio (HR)=2.02; 95% CI, 1.63–2.49; *P*<0.001) and overall survival (OS) (HR=1.91; 95% CI, 1.59−2.29; *P*<0.001) in GC, which was supported by Kaplan–Meier plotter analysis (www.kmplot.com), using microarray data from 876 GC patients^18^ (Figure 1f).

### Functional roles of E2F1 as a tumor activator *in vitro* and *in vivo*

**Figure 2:**
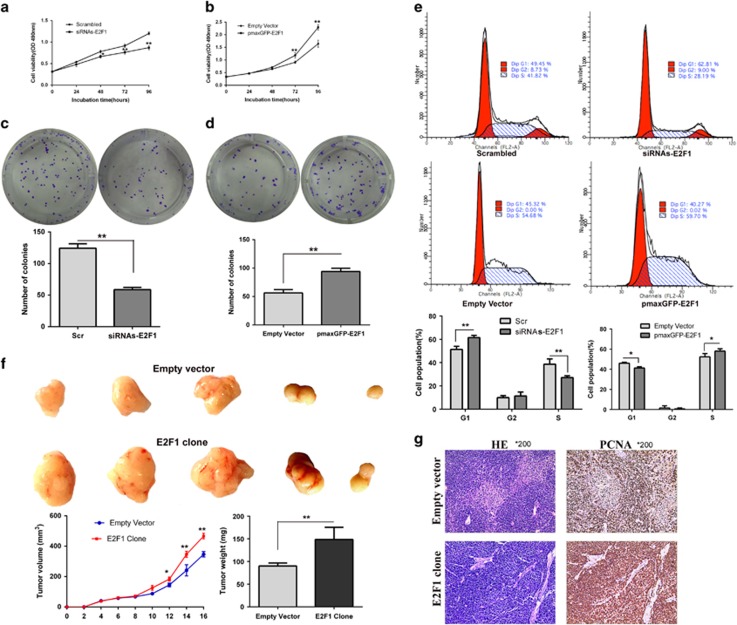
Functional roles of E2F1 *in vitro* and *in vivo*. E2F1 knockdown in GC cells transfected with siRNAs against E2F1 or E2F1 upregulation by pmaxGFP-E2F1 vector. E2F1 depletion inhibits GC cell growth, as detected by the (**a**) MTT assay and (**c**) colony-formation assay, whereas ectopic expression of E2F1 promotes GC cell growth, as examined by the (**b**) MTT assay and (**d**) colony-formation assay. Bars: S.D.; *P<0.05, **P<0.01. (**e**) Cell cycle analyses in the BGC823 and MGC803 cell lines. Relative to scrambled siRNA-transfected cells, E2F1 knockdown induced significantly increased the number of cells in the G0/G1 phase and reduced the number of cells in the S phase. Relative to empty vector-transfected cells, E2F1 upregulation promotes cell cycle progression. Representative FACS images and statistics based on three independent experiments. Bars: S.D.; *P<0.05, **P<0.01. (**f**) Representative data showed that overexpression of E2F1 significantly promote tumor growth in nude mice xenograft model. MGC803 cells were transfected with empty vector or E2F1 expression vector and then injected into mouse flanks. Tumor growth was measured every 2 days after injection, and tumors were harvested at day 16 and weighed. (**g**) Detection of the cell proliferation markers PCNA in xenograft tumors by IHC

To elucidate whether E2F1 could have a role in accelerating GC progression, gain- and loss-of-function approaches were used to evaluate the biological function of E2F1 in GC cell lines. We used chemically synthesized small interfering RNAs (siRNAs) to knockdown endogenous E2F1 in BGC823, which have relative high E2F1 expression. In addition, E2F1 was overexpressed by transfecting the pmaxGFP-E2F1 vector into MGC803 cell lines, which have relative low E2F1 expression. The depletion and ectopic expression of E2F1 in cells was confirmed by western blot (Supplementary Figure S1A). MTT ((3-(4, 5-dimethylthiazol-2-yl)-2, 5-diphenyltetrazolium bromide) tetrazolium) and colony formation assays revealed that cells transfected with siRNAs but not scrambled in BGC823, had significantly inhibited growth and proliferation of GC cells (Figures 2a and c). Meanwhile, ectopic overexpression of E2F1 by transfecting the MGC803 cell lines with the pmaxGFP-E2F1 vector, selected by the addition of G418, significantly promoted GC cell proliferation *in vitro* (Figures 2b and d). We also examined the effects of E2F1 on GC cell cycle progression. As illustrated in Figure 2e, inhibition of E2F1 markedly blocked the cell cycle at the G1–S phase, whereas overexpression of E2F1 promotes cell cycle progression. We extended the study of the E2F1 growth promotion role to *in vivo* athymic (nu/nu) mouse models, the results showed that E2F1-transfected cells developed significantly larger tumors than empty vector-transfected cells (Figure 2f). IHC staining analyses showed that alteration of E2F1 expression significantly changed the expression of the cell proliferation markers proliferating cell nuclear antigen (PCNA) in gastric cells (Figure 2g).

### E2F1 upregulate *TINCR* expression in GC cells

**Figure 3:**
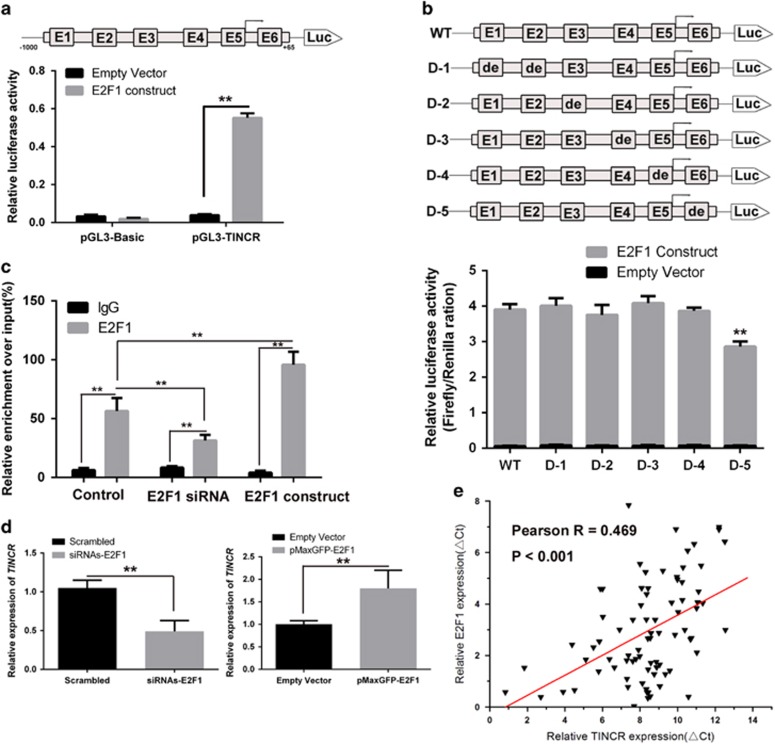
E2F1 upregulate *TINCR* expression in GC cells. (**a**) A dual-luciferase reporter assay was performed by co-transfection of the TINCR promoter fragment (TINCR-pGL3) with overexpression of E2F1. (**b**) Reporter assay in cells transfected with various *TINCR* promoters constructs with deletion in different binding elements for E2F1 (WT, wild type; D, deletion type). Luciferase activity was expressed as relative to that of the pGL3 vector (a promoter-less vector) (**c**) ChIP assay demonstrated endogenous E2F1 binding to the *TINCR* gene promoter, and the ectopic expression or siRNA knockdown, respectively, increased or reduced E2F1 enrichment on the *TINCR* promoter *P<0.05, **P<0.01. (**d**) qPCR analysis of *TINCR* expression levels following the treatment of BGC823 and MGC803 cells with siRNA-E2F1 and pmaxGFP-E2F1 expression vector, respectively. Bars: S.D.; *P<0.05, **P<0.01. (**e**) Analysis of the relationship between *TINCR* expression (ΔCt value) and E2F1 mRNA level (ΔCt value) in 80 GC tissues

Accumulating data revealed that E2F1 promote cancer progression by activation transcription of downstream oncogene in both coding and non-coding regions of the genome. Our previous study identified a lncRNA, *TINCR*, promotes GC proliferation and overexpression of *TINCR* indicates worse prognosis of GC. To unravel whether *TINCR* was regulated by E2F1 expression in GC, we examined the *TINCR* core promoter region for transcription factor binding sites, and identified six tandem putative E2F1-binding sites at the regions −366 to −355 bp (E1), −257 to −239 bp (E2), −136 to −124 bp (E3), −41 to −30 bp (E4), −16 to 0 (E5) and +56 to +73 bp (E6) in the *TINCR* promoter (Figure 3a). We cloned the human *TINCR* promoter fragment (nucleotides −1000 to +163) into pGL3 vector for a luciferase activity assay. *TINCR* transcriptional activity was induced by E2F1 overexpression (Figure 3a). The results suggested that E2F1 participate in *TINCR* transcription regulation. To validate this finding, we deleted these binding sites individually and used them repeated as the reporter assay. The results showed that the deletion of the E2F1-binding motif E6 significantly impaired the effect of E2F1 on *TINCR* transcription activation, suggesting that E2F1 binds to their special binding motifs to regulate *TINCR* transcription (Figure 3b). To corroborate this notion, we performed *in vivo* chromatin immunoprecipitation (ChIP) assays to address whether E2F1 bind to the *TINCR* promoter region. The ChIP assay revealed that endogenous E2F1 bound to the *TINCR* promoter (Figure 3c). We next determined whether the overexpression of *TINCR* is mediated by E2F1, we applied loss- and gain-of-function approaches. We showed that the ectopic expression or siRNA knockdown, respectively, increased or reduced E2F1 enrichment on the *TINCR* promoter (Figure 3c), and resulted, respectively, in *TINCR* upregulation or downregulation in GC cells (Figure 3d). The correlation of E2F1 and *TINCR* gene transcription were further elucidated in tissues sample, and the result revealed that of *TINCR* expression is positively correlated with E2F1 mRNA levels in GC (Pearson R=0.469, *P*<0.001) (Figure 3e). Hence, these results suggest that E2F1 serve as the transcriptional factors to activate *TINCR* transcription and upregulate its expression.

### Overexpression of *TINCR* is potentially involved in the tumor promotion function of E2F1

**Figure 4:**
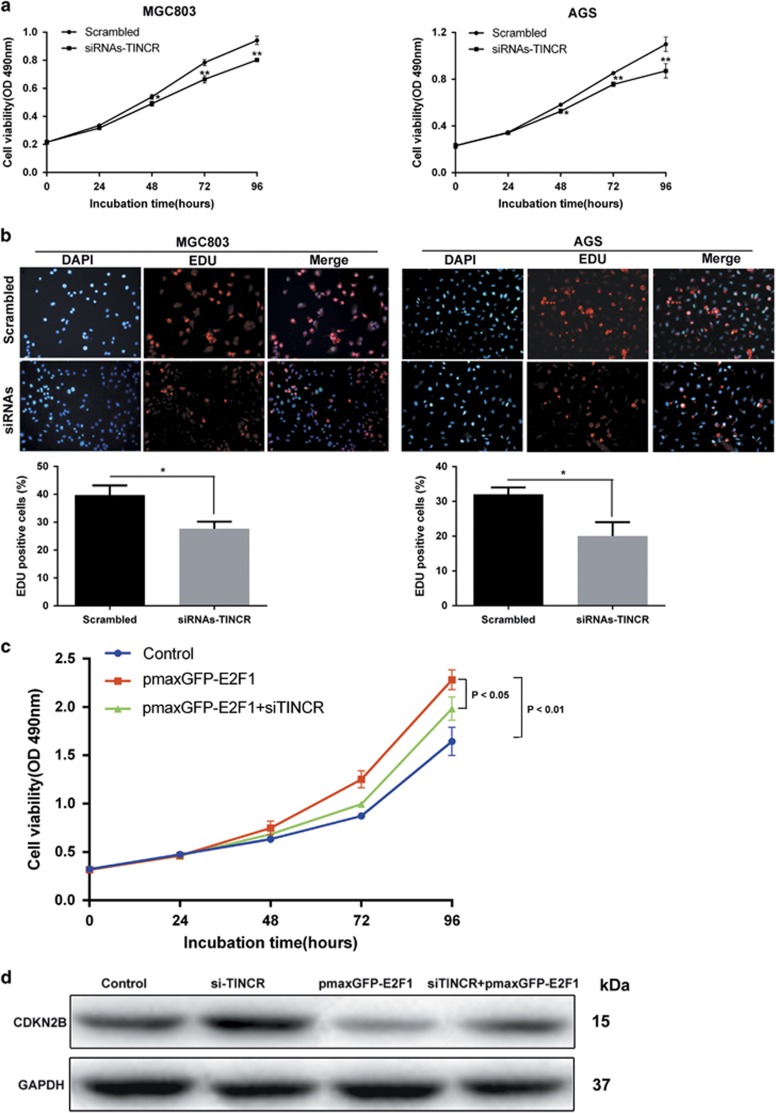
*TINCR* promotes cells proliferation and regulates *CDKN2B* expression in MGC803 and AGS cells; and is involved in the E2F1-mediated promotion of viability. (**a**) MTT assays were performed to determine the cell viability of siRNAs-*TINCR*-transfected GC cells. (**b**) EDU (red)/DAPI (blue) immunostaining assay was used to confirm the results of MTT assay. The data represent the mean±S.D. from three independent experiments. *P<0.05, **P<0.01. (**c**) The simultaneous depletion of *TINCR* could partly 'rescue' the proliferation effects induced by overexpressed E2F1 in MGC803 cells. Error bars represent S.D., *n*=3. *P<0.05; **P<0.01. (**d**) *TINCR* depletion upregulates the expression of CDKN2B in GC cells, and the simultaneous overexpression of E2F1 could partly 'rescue' the CDKN2B expression. The expression of CDKN2B protein in MGC803 cells was analyzed by western blotting

Our previous work found that *TINCR* could promote GC cell line BGC823 and SGC7901 proliferation.^17^ Here, we further confirm the result in MGC803 and AGS cell lines. We used chemically synthesized siRNAs to knockdown endogenous *TINCR* in MGC803 and AGS cell lines, which both were considered appropriate for *TINCR* depletion (Supplementary Figure S1B). MTT assays show that siRNA transfection-mediated *TINCR* knockdown resulted in a significant decrease in cell viability rate in MGC803 and AGS, which tend to exhibit naturally high *TINCR* expression levels (Figure 4a). These observations were further confirmed by EDU (red)/DAPI (blue) immunostaining assay (Figure 4b). To investigate whether *TINCR* was involved in the E2F1-induced increase in GC cell proliferation, we carried out rescue experiments. After transfection with si-*TINCR*, MGC803 cells were co-transfected with pmaxGFP-E2F1. MTT assays indicated that the co-transfection could partially rescue pmaxGFP-E2F1-promoted proliferation in MGC803 cells. (Figure 4c). Moreover, we found that co-transfection of pmaxGFP-E2F1 could rescue the upregulated expression of *CDKN2B* protein induced by the depletion of *TINCR* (Figure 4d). These data indicated that E2F1 promotes GC cell proliferation partly through the upregulation of *TINCR* expression.

### *TINCR* targets *CDKN2B* by SMD

Our previous study revealed that most *TINCR* molecules are located within the cytoplasm, and are bound to STAU1 protein in GC cells, and the results are further confirmed in MGC803 and AGS cell lines (Supplementary Figure S2). KLF2 mRNA was detected as a bona fide SMD target, which was mediated by *TINCR* in GC cells in our recent publication. We hypothesized that *CDKN2B,* which was elevated upon *TINCR* depletion, may also be direct *TINCR*-STAU1 complex targets. First, we analyzed the RNA interactome analysis data followed by deep sequencing (RIA-sequencing) provided by online GEO data sets (http://www.ncbi.nlm.nih.gov/geo/query/acc.cgi?acc=GSE40121), and found that *CDKN2B* are also bound to *TINCR* mRNA (Supplementary Table S3). And the binding regions are located at the 3′-UTR region of *CDKN2B* mRNA (Supplementary Figure S3A). Previous evidence confirmed that *TINCR* interacts with mRNA through a 25-nucleotide motif that was strongly enriched in *TINCR*-interacting mRNAs and also repeated within *TINCR* itself, termed the *TINCR* box (Supplementary Figure S3B).^19^ We also speculated the *CDKN2B* sequence bound to the *TINCR* box (Supplementary Figure S3C).

**Figure 5:**
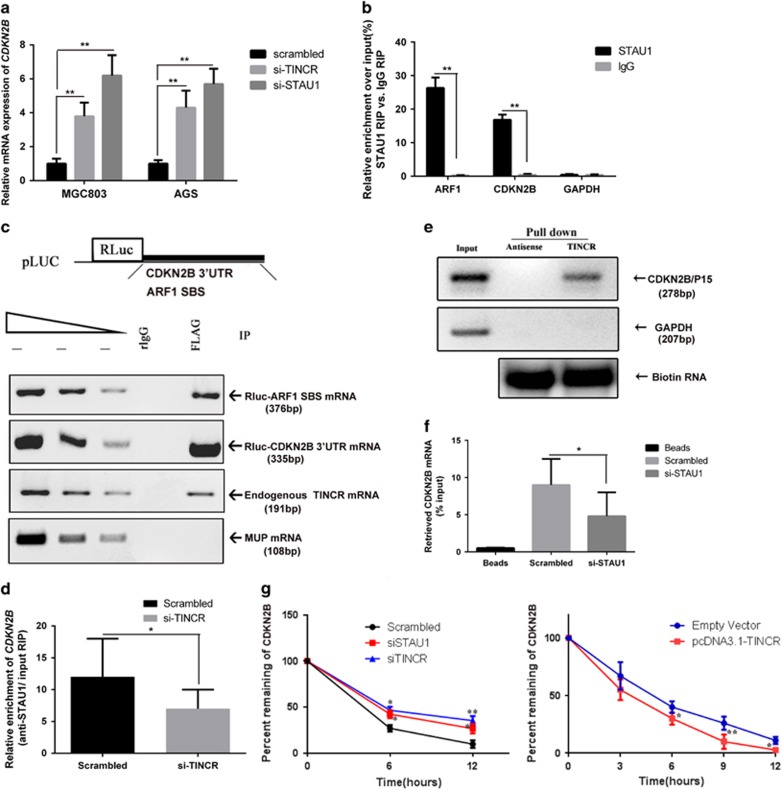
*TINCR*-STAU1 complex binds to *CDKN2B* mRNA and regulates their stability. (**a**) The abundance of *CDKN2B* mRNA was elevated upon *TINCR* and STAUI depletion in GC cells, detected by qRT-PCR. Error bars represent S.D., *n*=3. **P<0.01. (**b**) Interaction of *CDKN2B* mRNA with STAU1, detected by RIP assay (the relative ARF1 enrichment served as a positive control, and the GAPDH as a negative control that did not interact with STAU1). Error bars represent S.D., *n*=3. **P<0.01. (**c**) IP of STAU1-FLAG. MGC803 cells were transiently co-transfected with (1) STAU1-FLAG expression plasmid; (2) RLuc-CDKN2B 3′-UTR; (3) phCMV-MUP, which encodes MUP mRNA that lacks an SBS and serves as a negative control for STAU1-FLAG binding; and (4) Rluc-ARF1 SBS, which contains an ARF1 SBS downstream of the translation termination codon of C-terminally deleted renilla luciferase and serves as a positive control for STAU1-FLAG binding. After cell lysis, total RNA and protein were purified from the lysate before and after IP using FLAG antibody or nonspecific rabbit (r) IgG. The three leftmost lanes represent two-fold serial dilutions of RNA and demonstrate that the RT-PCR is semiquantitative. Schematic representations of the pLUC-*CDKN2B* 3′-UTR and pLUC-ARF1 SBS test plasmids (above). RT-PCR analysis demonstrates that *CDKN2B* 3′-UTRs, endogenous *TINCR,* and ARF1 SBS bind STAU1-FLAG, whereas MUP mRNA does not (below). Results are representative of three independently performed experiments. (**d**) Inhibiting *CDKN2B* mRNA interacting with STAU1 upon *TINCR* depletion, detected by RIP experiments. MGC803 cells were transfected with control (Scrambled) or si-*TINCR*, and cellular extract was prepared for RIP assay using SATU1 antibody 24 h after transfection. Error bars represent S.D, *n*=3. *P<0.05. (**e**) Biotinylated *TINCR* RNA pulls down the full-length *CDKN2B* mRNA detected by RT-PCR analysis. A nonspecific RNA (GAPDH) is shown as a control. (**f**) STAU1 depletion reduced the interaction between *TINCR* with *CDKN2B* mRNA. MGC803 cells were transfected with control (Scrambled) or si-STAU1, and cell lysates were incubated with biotin-labeled *TINCR*; after pull-down, mRNAs were extracted and assessed by qRT-PCR. Error bars represent S.D., *n*=3.*P<0.05; **P<0.01. (**g**) *TINCR* or STAU1 control *CDKN2B* mRNA stability. RNA stability assays were performed in MGC803 cells using Actinomycin D to disrupt RNA synthesis degradation rates of the mRNA *CDKN2B* over 12 h. *P<0.05; **P<0.01

In order to confirm the above speculation, we performed *in vitro* assays in GC cells. First, we knockdown endogenous *TINCR* and STAU1 in GC cells, which both were considered appropriate depletion (Supplementary Figure S1B and C), and the abundance of *CDKN2B* mRNA increased upon *TINCR* and STAU1-depleted GC cells (Figure 5a). Second, RNA immunoprecipitation (RIP) assays showed a remarkable enrichment of *CDKN2B* by STAU1 antibody compared with IgG control, indicating STAU1 could bind to *CDKN2B* mRNA (Figure 5b). Third, we determined whether the binding regions are located in 3′-UTRs, and cells were transfected with these test plasmids: pLUC-*CDKN2B* 3′-UTR, the STAU1-FLAG expression vector, pLUC-*ARF1* SBS, and phCMV-MUP reference plasmid, which encodes major urinary protein (MUP) mRNA. The two latter of these served as a positive and a negative control, respectively, for STAU1-FLAG binding.^16^ Anti-FLAG could immunopurifiy Rluc-*CDKN2B* 3′-UTR, endogenous *TINCR* and Rluc-*ARF1* SBS, but not MUP mRNA (Figure 5c). Those results indicate that *CDKN2B* is a bona fide SMD target in GC cells.

To further determine whether *TINCR* is required for the co-IP of STAU1 with *CDKN2B* mRNA, MGC803 cells that transiently transfected with control siRNA or siRNA against *TINCR* were immunoprecipitated using anti-STAU1 antibody. Compared with control siRNA, siRNA-*TINCR* reduced by ~2-fold the co-IP of STAU1 with *CDKN2B* mRNA (Figure 5d). Furthermore, the RNA pull-down assay revealed that *TINCR* interacted with *CDKN2B* mRNA (Figure 5e), and the depletion of STAU1 significantly reduced the interaction of *TINCR* with *CDKN2B* mRNA (Figure 5f), corroborating that STAU1 is required for the association between *TINCR* and *CDKN2B* mRNA. More importantly, the *CDKN2B* mRNA half-life was significantly increased upon downregulation of STAU1 or *TINCR*, whereas it was decreased after *TINCR* overexpression (Figure 5g). Our findings suggest that *TINCR* affects *CDKN2B* mRNA stability and expression through SMD.

## Discussion

Recent findings have suggested that E2F family proteins have important roles in human malignancies.^10^ E2F1, a key regulator for the G1/S phase transition in the E2F family,^20^ was reported to upregulate in GC.^14^ However, the function role in GC progression remains disagreed. In this study, we found that E2F1 expression was significantly upregulated in GC tissues compared with corresponding non-cancerous tissues. Specifically, E2F1 expression levels could be used to discriminate the cancer tissues from non-tumorous tissues. Moreover, patients with higher E2F1 levels appeared to have a greater tumor size, higher tumor stage and shorter survival than the lower group. Our results indicate that E2F1 expression provided a significantly predictive value and prognostic marker for patients with GC.

Our data revealed that silencing E2F1 expression led to significant inhibition of cell proliferation, whereas E2F1 overexpression contributed to cell growth and tumorigenicity. Knockdown of E2F1 expression contributed to G1 phase arrest and an S phase reduction, whereas ectopic overexpression of E2F1 promoted cell cycle progression. Accumulation data revealed that E2F1 exert cell cycle modulation function by regulation of both coding and non-coding transcripts. A novel lncRNA, named *TINCR*, a potent cell cycle modulator in GC was identified in our recent work. Hence, we speculate that E2F1 and *TINCR* occurrence of mutual reaction. In this study, we found E2F1 could bind around +56 to +73 bp of *TINCR* promoter region and specifically activated its transcription. The G1–S transition in the cell cycle in mammalian cells is controlled by cyclins, cyclin-dependent kinases (CDKs), and their inhibitors, and deregulation of CDK inhibitors is a common feature in tumor cells.^21^ *CDKN2B* serve as potent growth inhibitors of cell cycle checkpoints.^21^ Notably, consistent with our recent report, *CDKN2B* was found to be remarkably upregulated upon *TINCR* or E2F1 knockdown in MGC803 and AGS cells. Taken together, *CDKN2B* could be crucial *TINCR* and E2F1 target.

**Figure 6:**
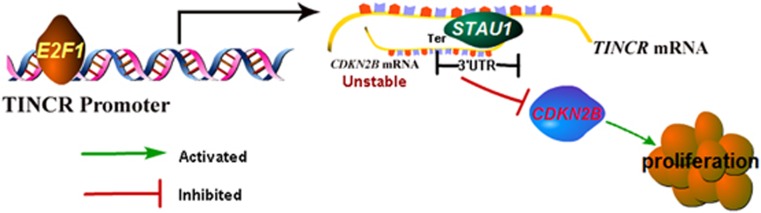
Summary diagram describes that E2F1 and *TINCR* regulates GC cell proliferation

LncRNAs can act together with specific proteins to perform various functions depending on their subcellular location,^22, 23^ and *TINCR* is a predominantly cytoplasmic lncRNA in GC cells, indicating its action in post-transcriptional gene regulation. The results of RNA IP and RNA pull-down assays show that *TINCR* could bind STAU1, which is consistent with our previous data.^17^ STAU1 is a cytoplasmic protein and exerts multiple effects as a post-transcriptional regulator. Our teams have identified that *TINCR* targets *KLF2* transcript through *TINCR*–STAU1 complex formation. Here, this study found that *CDKN2B* is also a target of STAU1. In addition, *CDKN2B* mRNA stability and the effects of binding to STAU1 are influenced by *TINCR* depletion. As evidenced above, *TINCR* may affect *CDKN2B* expression through SMD by *TINCR*-STAU1 complex formation. The pathway via which E2F1 and *TINCR* regulate cell cycle and cells proliferation has been depicted in Figure 6. The nuclear transcription factor E2F1 induces *TINCR* overexpression. *TINCR* recruits STAU1 to the 3′-UTR of *CDKN2B* mRNA, degrading *CDKN2B* through the UPF1-dependent mRNA decay mechanism. Subsequently, *CDKN2B* depletion promotes cell cycle progression and tumorigenicity. Here, we explored a novel pathway involved in E2F1, *TINCR* and *CDKN2B* in GC development.

We describe here a novel mechanism underlying GC cell proliferation through a molecular cross talk between E2F1*, TINCR*, STAU1 and CDKN2B. Further insights into the functional and clinical implications of the pathway may contribute to early GC diagnosis and help with GC treatment.

## Materials and Methods

### Plasmids, RNA interference and transfection

pmaxGFP-E2F1 expression vector was purchased from Addgene (plasmid #16007) (Cambridge, USA). To construct Rluc-*CDKN2B* 3′-UTR and Rluc-ARF1 SBS, pLuc luciferase vector (Ambion Inc., Grand Island, NY, USA) carrying the *Renilla* luciferase (Rluc) reporter gene was digested with an endonuclease and ligated to the corresponding fragment that encodes human *CDKN2B* 3′-UTR and ARF1 SBS mRNA. The *CDKN2B* 3′-UTR and ARF1 SBS mRNA fragments were amplified by PCR using a cDNA library from MGC803 cells as a template and the primers listed below: for *CDKN2B* 3′-UTR: 5′-cacaa**ctcgag**CACCCCCACCCACCTAATTC-3′ (sense) and 5′-tga*agatct*TGCCAGGTGGCTTCGAAAAT-3′ (antisense), where the bold nucleotides specify the *Xho*I site, and the italic underlined nucleotides specify the *Bgl*II site.

For ARF1 SBS mRNA: 5′-cacaa**gtcgac**GTGAACGCGACCCCCCTCCCTCTCACTC-3′ (sense) and 5′-aa*ggatcc*CCAGGTGCCCATGGGCCTACATCCCC-3′ (antisense), where the bold nucleotides specify the *Sal*I site, and the italic nucleotides specify the *Bam*HI site. To construct the luciferase reporter vectors, the core promoter of the *TINCR* gene (−1000 to +163, relative to the transcription start site of the *TINCR* gene) and the relative deletion of binding sites were respectively subcloned into the pGL3 basic firefly luciferase reporter. siRNAs for specifically knockdown E2F1, *TINCR* and STAU1 were chemically synthesized (Invitrogen, Shanghai, China), and the sequences of the oligonucleotides synthesized for RNAi have been listed in Supplementary Table S1. Transfections were carried out using Lipofectamine 2000 reagent according to the manufacturer’s instructions (Invitrogen, Shanghai, China).

### Cell lines and immunoblot analysis

The human gastric adenocarcinoma cancer cell lines MGC803, BGC823, MKN45, AGS and SGC7901 and the normal gastric epithelium cell line (GES-1) were obtained from the Chinese Academy of Sciences Committee on Type Culture Collection Cell Bank (Shanghai, China). Western blot analysis was conducted according to our previous protocol.^24^ Antibodies used in the study were: E2F1 (cat. # ab14768, Abcam, Hong Kong, China), CDKN2B (cat. # sc-271791, Santa Cruz, Dallas, TX, USA), STAU1 (03-116, Millipore, Bedford, MA, USA), FLAG-tagged antibodies (8146 S, Cell Signaling Technology, Boston, MA, USA), and GAPDH antibody was used as control.

### Tissue samples and clinical data collection

In this study, 80 patients underwent primary GC resection at the First Affiliated Hospital of Nanjing Medical University and the Affiliated Hospital of Yangzhou University. The study was approved by the ethics committee on Human Research of the First Affiliated Hospital of Nanjing Medical University and the Affiliated Hospital of Yangzhou University. Written informed consent was obtained from all patients. The clinicopathological characteristics of the GC patients have been summarized in Supplementary Table S2.

### RNA preparation and quantitative real-time PCR

Total RNAs were extracted with TRIzol reagent (Invitrogen, Grand Island, NY, USA), and quantitative real-time PCR (qRT-PCR) analyses were conducted according to the manufacturer’s instructions (Takara, Dalian, China). The primers sequences have been listed in Supplementary Table S1.

### Isolation of cytoplasmic, and nuclear RNA

Cytoplasmic and nuclear RNA were isolated and purified using the Cytoplasmic & Nuclear RNA Purification Kit (Norgen, Belmont, CA, USA), according to the manufacturer’s instructions.

### IHC analysis

To quantify protein expression, both the intensity and extent of immunoreactivity were evaluated and scored. In the present study, staining intensity was scored as follows: 0, negative staining; 1, weak staining; 2, moderate staining; and 3, strong staining. The scores of the extent of immunoreactivity ranged from 0 to 3, and were determined according to the percentage of cells that showed positive staining in each microscopic field of view (0, <25% 1, 25–50% 2, 50–75% 3, 75–100%). A final score ranging from 0 to 9 was achieved by multiplying the scores for intensity and extent. Using this method, the expression of proteins was scored as 0, 1, 2, 3, 4, 6 or 9. In case of disagreement (score discrepancy 0.1), slides were reexamined and a consensus was reached by the experts.

### Luciferase reporter assay

Cells were first transfected with appropriate plasmids in 24-well plates. Next, the cells were collected and lysed for luciferase assay 48 h after transfection. The relative luciferase activity was normalized with *Renilla* luciferase activity.

### Cell proliferation assays

Cell proliferation assays and colony formation assays were performed as previously reported.^24^

### Flow cytometry

Cell cycle and cell apoptosis were analyzed by flow cytometry and detected as previously reported.^24^

### EDU analysis

5-Ethynyl-2-deoxyuridine (EDU) labeling/detection kit (Ribobio, Guangzhou, China) was used to assess the cell proliferation. Cells were grown in 96-well plates at 5 × 103 cells per well. Forty-eight hours after transfection, 50 *μ*M EdU labeling media were added to the 96-well plates and they were incubated for 2 h at 37 °C under 5% CO_2_. After treatment with 4% paraformaldehyde and 0.5% Triton X-100, cells were stained with anti-EdU working solution. DAPI was used to label cell nuclei. The percentage of EdU-positive cells was calculated after analyses of fluorescent microscopy. Five fields of view were randomly assessed for each treatment group.

### Chromatin immunoprecipitation

ChIP assays were performed using the EZ ChIPTM Chromatin Immunoprecipitation Kit (Millipore), according to the manual. The primer sequences were listed in Supplementary Table S1.

### RIP and RNA pull-down

We performed RIP experiments using the Magna RIP RNA-Binding Protein Immunoprecipitation Kit (Millipore) according to the manufacturer’s instructions. The STAU1 and FLAG-tagged antibodies used for IP were from Millipore (03-116; RIPAb+ STAU1) and Cell Signaling Technology (8146S), respectively. The details of the primers for RT-PCR and qPCR have been provided in Supplementary Table S1.

Biotin-labeled RNAs were transcribed *in vitro* with the Biotin RNA Labeling Mix (Roche Diagnostics, Shanghai, China) and T7 RNA polymerase (Roche Diagnostics), treated with RNase-free DNase I (Roche Diagnostics) and purified with an RNeasy Mini Kit (Qiagen, Valencia, CA, USA). Next, 1 mg whole-cell lysates from MGC803 cells was incubated with 3 *μ*g of purified biotinylated transcripts for 1 h at 25 °C. Complexes were isolated with streptavidin agarose beads (Invitrogen, Grand Island, NY, USA). The beads were washed briefly three times and boiled in sodium dodecyl sulfate buffer, and the retrieved protein was detected using the standard western blot technique. The RNA present in the pull-down material was detected using reverse transcription polymerase chain reaction (RT-PCR) and qPCR analysis. The RT-PCR and qPCR primer pairs were provided in Supplementary Table S1.

### RNA stability assay

To analyze RNA stability, GC cells were treated with actinomycin D (1 *μ*g/ml). Cells were collected at different time points, and RNA was extracted using Trizol reagent (Invitrogen, Grand Island, NY, USA). Reverse transcription was performed using oligo (dT) primers and mRNA levels were measured using qRT-PCR.

### Bioinformatics analysis and statistical analysis

GC gene expression data was obtained from the NCBI GEO, (http://www.ncbi.nlm.nih.gov/geo/). One data set GSE51575 consisted of 26 paired primary gastric adenocarcinoma tissues and surrounding normal fresh frozen tissues was included. All the tissues were obtained after curative resection and pathologic confirmation at Samsung Medical Center (Korea cohort). The raw CEL files from the Agilent arrays (Agilent, Santa Clara, CA, USA) for GSE51575 were processed and normalized using the Robust Multichip Average as previously described.^25^ All statistical analyses were performed using SPSS 20.0 software (IBM, SPSS, Chicago, IL, USA). The significance of differences between groups was estimated using the Student’s *t*-test, *χ*2 test, Fisher’s exact test, Mann-Whitney test, Kruskal–Wallis test or Wilcoxon test, as appropriate. A ROC curve was established to evaluate the diagnostic value for differentiating between GC and benign diseases. FP survival (FPS) and OS rates were calculated by the Kaplan–Meier method with the log-rank test applied for comparison. Pearson correlation analysis was performed to investigate the correlation between *TINCR* and *E2F1* mRNA expression. Two-sided *P*-values were calculated, and a probability level of 0.05 was chosen for statistical significance.
