# Supplementary figures and images for: E2F1 induces TINCR transcriptional activity and accelerates gastric cancer progression via activation of TINCR/STAU1/CDKN2B signaling axis
Source: Cell Death Dis. 2017 Jun 1;8(6):e2837–. doi: 10.1038/cddis.2017.205 (PMC5520882; doi:10.1038/cddis.2017.205)

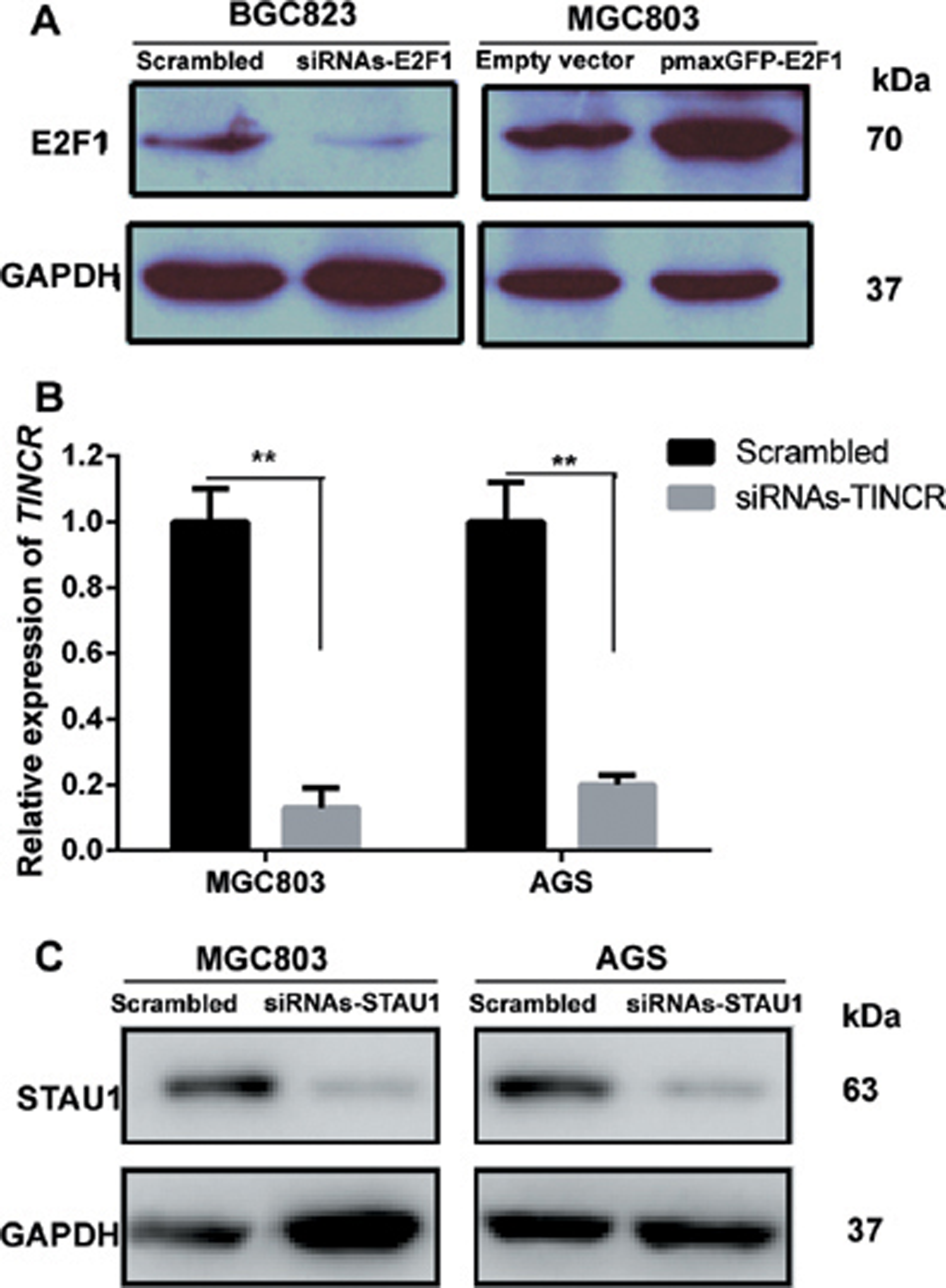

Supplement: Supplementary Figure S1 [file cddis2017205x1.tif]

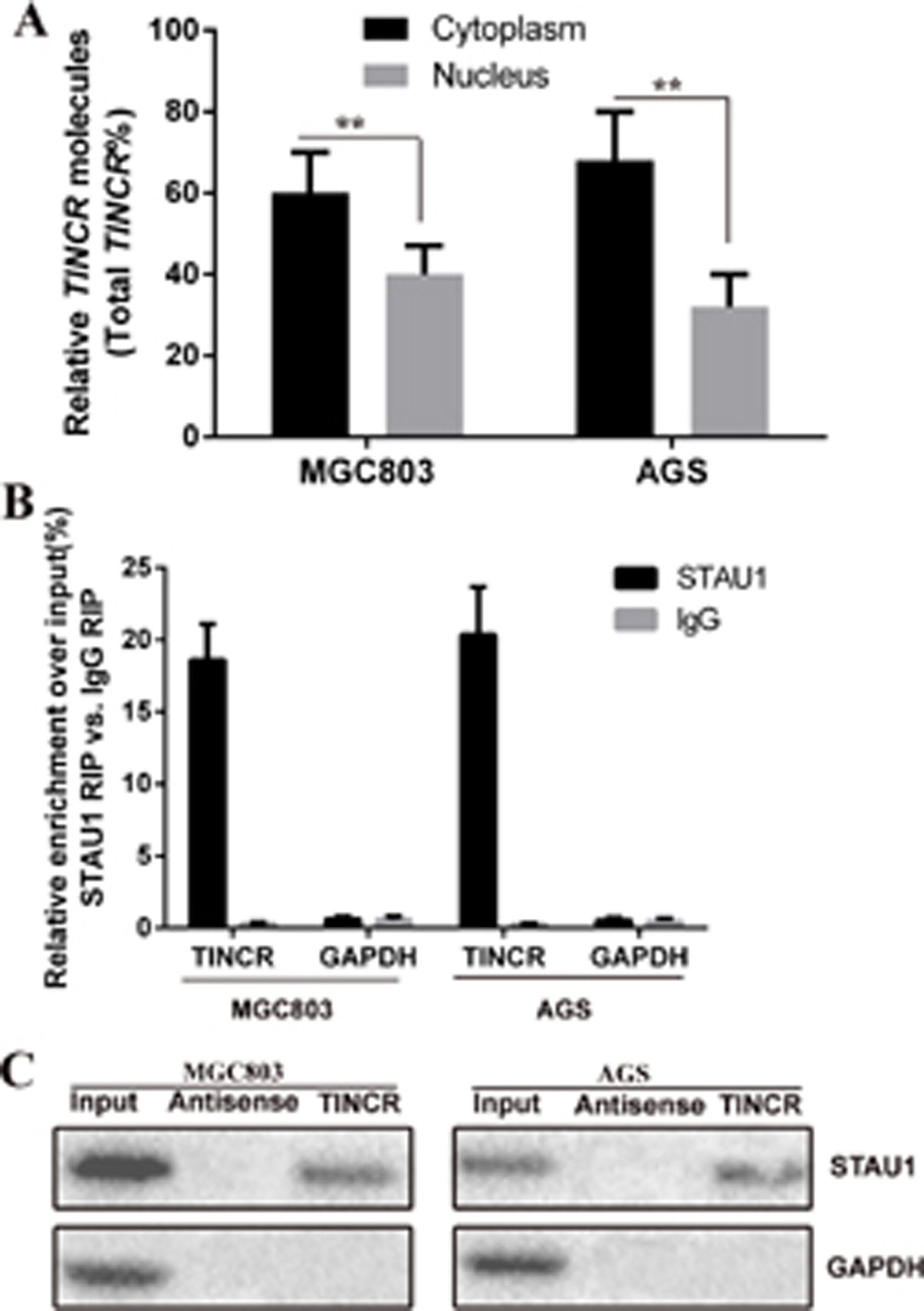

Supplement: Supplementary Figure S2 [file cddis2017205x2.tif]

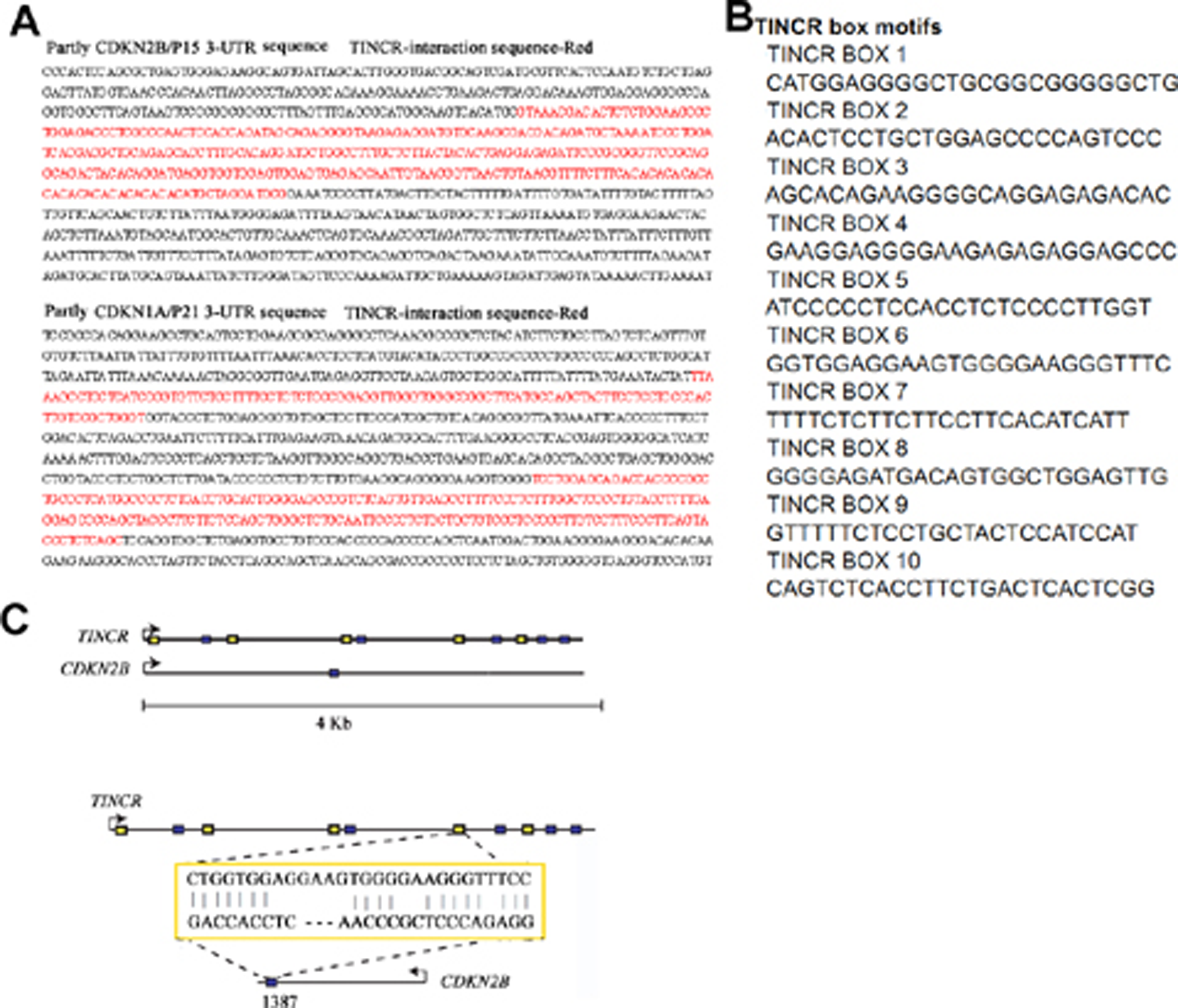

Supplement: Supplementary Figure S3 [file cddis2017205x3.tif]
