# Supplementary material for: E2F1 induces TINCR transcriptional activity and accelerates gastric cancer progression via activation of TINCR/STAU1/CDKN2B signaling axis
Source: Cell Death Dis. 2017 Jun 1;8(6):e2837–. doi: 10.1038/cddis.2017.205 (PMC5520882; doi:10.1038/cddis.2017.205)
Supplement: Supplementary Legends [file cddis2017205x7.docx]

**Figure S1.** Analysis of E2F1, *TINCR,* and STAU1 expression levels following the treatment of GC cells with siRNAs-E2F1 or pmaxGFP-E2F1 expression vector, siRNAs-*TINCR*, and siRNAs-STAU1

(A) BGC823 cells were transfected with siRNAs against E2F1 (siRNA)/scramble-control siRNA (negative control), and MGC803 cells were transfected with pmaxGFP-E2F1 vector/Empty vector (negative control), respectively, for 48 h in cells. Reduced E2F1 expression by siRNAs or elevated E2F1 expression by pmaxGFP-E2F1 vector was shown by western blotting analysis normalized to GAPDH. (B) *TINCR* transcript expression was quantiﬁed by qRT-PCR after treatment with si-*TINCR* (siRNAs)/scramble-control siRNA in MGC803 or AGS cells. Error bars represent SD, n = 3. ***P* < 0.01. (C) STAU1 expression was detected by western blot after treatment with si-STAU1 (siRNAs)/scramble-control siRNA in MGC803 or AGS cells.

**FigureS2** *TINCR* interacts with cytoplasmic protein STAU1

**(A)** Subcellular location of *TINCR* detected by the protocol of isolating nuclear RNA and cytoplasmic RNA, indicating *TINCR* mainly locates in cytoplasm of MGC803 and AGS cells. **(B)** Interaction of *TINCR* mRNA with STAU1. RIP experiments were performed using the STAU1 antibody to immunoprecipitate (IP) in total cell extracts of MGC803 and AGS cells, and relative enrichment was determined as RNA associated with STAU1 IP relative to an input control, GAPDH as a negative control that do not interact with STAU1. Error bars represent SD, n = 3. ***P* < 0.01. **(C)** Biotinylated *TINCR* or antisense RNA was incubated with total cell extracts (MGC803 and AGS cells), targeted with streptavidin beads, and washed; the associated proteins were resolved in a gel. Western blotting analysis of the speciﬁc association of STAU1 with *TINCR* (n = 3). A nonspeciﬁc protein (GAPDH) is shown as a control.

**FigureS3** *TINCR* interacts with *CDKN2B* mRNA based on bioinformatics analysis

**(A)** The highlighted parts of the *CDKN2B* sequences interact with *TINCR* (red), identified from data of previously published RIA-sequencing ([Kretz et al., 2013](#_ENREF_14)). **(B)** Table depicting the 10 *TINCR* box motifs present in the *TINCR* transcript, which has been identified in a previous report ([Kretz et al., 2013](#_ENREF_14)). **(C)** *TINCR* motif base-pairing between the *TINCR* transcript and *CDKN2B* mRNA.

**Table S1**. Primers used for qRT-PCR, RT-PCR, qChIP, and siRNAs oligonucleotides.

**Table S2**. Clinical data of all patients; *TINCR* expression (relative to GAPDH) and E2F1 relative mRNA expression (relative to GAPDH) and immunohistochemical sores.

**Table S3**. RNA interactome analysis with high throughput sequencing for *TINCR* (PLAC2).
